# Serological and hematological characteristics of Sjogren’s syndrome and dry eye syndrome patients using a novel immune serology technique

**DOI:** 10.1371/journal.pone.0244712

**Published:** 2020-12-31

**Authors:** Hadas Ben-Eli, Abraham Solomon, Doron J. Aframian, Eldad Ben-Chetrit, Dror Mevorach, Geffen Kleinstern, Tim Waterboer, Martina Willhauck-Fleckenstein, Michael Pawlita, Ora Paltiel

**Affiliations:** 1 Braun School of Public Health and Community Medicine, Hadassah-Hebrew University, Jerusalem, Israel; 2 Department of Ophthalmology Hadassah-Hebrew University Medical Center, Jerusalem, Israel; 3 Department of Optometry and Vision Science, Hadassah Academic College, Jerusalem, Israel; 4 Department of Oral Medicine, Sedation and Maxillofacial Imaging and Sjogren’s Syndrome Center, Hadassah-Hebrew University Medical Center, Jerusalem, Israel; 5 Unit of Rheumatology, Hadassah-Hebrew University Medical Center, Jerusalem, Israel; 6 Department of Internal Medicine, Hadassah-Hebrew University Medical Center, Jerusalem, Israel; 7 Department of Health Sciences Research, Mayo Clinic, Rochester, MN, United States of America; 8 Infections and Cancer Epidemiology, German Cancer Research Center (DKFZ), Heidelberg, Germany; 9 Department of Hematology, Hadassah-Hebrew University Medical Center, Jerusalem, Israel; Universita Cattolica del Sacro Cuore, ITALY

## Abstract

**Objectives:**

To compare hematologic and serological parameters among patients with Sjogren’s syndrome (SS), dry eye syndrome (DES) and controls, and validate a novel multiplex-serology method for identifying auto-antibodies in these populations.

**Methods:**

In a clinic-based case-control study a total of 422 participants were recruited, including 91 with SS, 120 DES, and 211 controls (age and sex frequency-matched). We measured blood counts, anti-nuclear-antibodies (ANA), anti-SSA/SSB, anti-ribonucleoprotein (RNP), anti-double-stranded-DNA (DS-DNA), and rheumatoid factor (RF) using the “*Immunodot”* qualitative-ELISA assay. Immunoglobulins, C3 and C4 were measured by immune-fluorescence. Autoantibodies were also quantified with a newly-developed method using glutathione-S-transferase fusion proteins of SSA/Ro 52 and 60kD and SSB/La (multiplex-serology), measuring median fluorescence intensity (MFI).

**Results:**

Among DES patients, only 2% (95%CI: 0.36–6.3) had positive immune serology. SS patients had lower lymphocyte, hemoglobin and C3 levels but higher prevalence of RF, ANA, anti-SSA/B and higher IgG and MFI levels, compared to DES and controls (P<0.001). Presence of anti-SSA/Ro-52kD was associated with SS [odds ratio (OR) = 2.05, 95% confidence interval (CI): 1.46–2.88]. Anti-SSB/La was inversely associated with DES (OR = 0.81, 95%CI: 0.65–1.00) compared to controls. Positivity to RF (adjusted for age, gender and ethnicity OR = 5.03, 95%CI: 1.78–14.21), ANA (OR = 14.75, 95%CI: 4.09–53.17), or combination of anti-SSA/B (OR = 20.97, 95%CI: 4.60–95.54) were more likely in SS compared to DES. The novel multiplex-serology method correctly identified anti-SSA/B autoantibodies by ELISA among SS, DES patients and controls (sensitivity = 1.0, negative-predictive-value = 1.0).

**Conclusions:**

Serologic parameters distinguish SS from DES patients and controls. A newly-developed multiplex-serology technique may be useful to detect autoantibodies in large epidemiologic studies.

## Introduction

Dry eye syndrome (DES), also named keratoconjunctivitis sicca (KCS), among the most frequent diagnoses in ophthalmology [1], is defined by the International Dry Eye Workshop (DEWS) as a “multifactorial disease of the tears and ocular surface that results in symptoms of discomfort, visual disturbance, and tear film instability with potential damage to the ocular surface” [2]. The prevalence of DES in the general population is estimated at 10–13.3% [3,4], and is highest in women and in the elderly, occurring in up to 67.3% of those aged over 71 [5]. These prevalence rates are probably greatly underestimated since many patients are self-treated and milder cases with intermittent symptomatology may not seek medical attention.

DES may also be a manifestation of Sjogren’s syndrome (SS) a systemic autoimmune disease (AID)—characterized by mononuclear lymphocytic infiltrations of the exocrine glands and epithelia affecting many organ systems [6]. SS can present as an entity in itself–primary SS (pSS)–or may be secondary (sSS) to other autoimmune conditions such as rheumatoid arthritis, systemic lupus erythematosus, systemic sclerosis and other AID [6]. Clinical presentations can vary considerably from relatively mild sicca symptoms, arthralgias, and fatigue to severe systemic symptoms such as vasculitis, glomerulonephritis, synovitis, interstitial lung disease, neuropathy, renal disease, auto-immune cytopenia and neurological manifestations [7]. Hematologic disturbances such as anemia, lymphocytopenia, neutropenia, and hypergammaglobulinemia [8,9] also occur. The heterogeneity and lack of specificity of signs and symptoms often leads to a delay in diagnosis [10]. The presence of serum autoantibodies, such as anti-SSA/Ro and anti-SSB/La, antinuclear antibodies (ANA) as well as positive rheumatoid factor (RF) are included in the diagnostic criteria of SS. SS patients can also manifest several hematologic disturbances such as anemia, lymphocytopenia, neutropenia, and hypergammaglobulinemia [11,12].

Estimates of SS prevalence in general populations range from 0.03% to 2.7% [6], varying according to classification criteria used, geographical region and ethnicity. The prevalence of SS in the Israeli population is unknown, and data regarding the proportion of patients with DES as an underlying manifestation of SS are lacking. In this study we aimed to estimate the prevalence of SS-related serological markers among DES patients and to compare hematologic parameters and immune serology among SS, DES and healthy controls using a standard, as well as a newly-developed immunologic method.

## Methods

### Study population

In a clinic-based case-control study, 422 Israeli participants were recruited, including 91 patients with established SS, 120 patients with established DES and 211 controls [13] frequency-matched by age and sex, as previously reported [14]. SS was diagnosed by a rheumatologist, cornea ophthalmologist or oral surgeon according to the American–European Consensus Group (AECG) classification [11], in which patients had to fulfill four out of six criteria: 1) subjective complaint of dry eyes; 2) complaint of xerostomia (dry mouth); 3) objective evidence of dry eyes; 4) objective diagnosis of xerostomia; 5) a positive minor salivary gland biopsy with a focus score of ≥1/4 mm^2^; 6) the presence of anti-SSA/Ro or anti-SSB/La antibodies. DES was diagnosed by a cornea ophthalmologist or by self-reported symptoms of dry eyes corroborated by a self-administered Ocular Surface Disease Index (OSDI) questionnaire score of >25 [15–17] and by Schirmer I test (without topical anesthesia), showing <10mm in 5 min or break of fluorescein corneal dye in < 5 sec in Tear Break Up Time (TBUT) of at least one eye among patients without other SS symptoms and did not fill the SS diagnostic criteria [18].

Since the Israeli population includes a high proportion of immigrants, ethnicity was grouped into five cardinal categories of East European, West Asian (mainly Iran, Iraq, Yemen), North African, Israeli or mixed origin, based on a score counting the number of relatives (mother, father, grandmothers and grandfathers) from the same world region (≥3). Participants were queried about first-degree relatives with AID, and SS patients reported their primary clinical presentations of joint pain, dry mouth and dry eyes.

#### Inclusion/Exclusion criteria

We recruited individuals aged ≥18 years. DES patients who had undergone ocular surgery in the five years preceding the date of enrollment or whose symptoms were due to chronic corneal infection or abscess were excluded. Other exclusion criteria were non-Israeli residency, refusal to sign or inability to understand the consent form or inability to understand any of the four questionnaire languages (Hebrew, English, Arabic or Russian). From the control group we excluded participants with a self-reported history of DES, OSDI score >25, SS, lymphoproliferative disease, and first-degree relatives of patients enrolled in the study.

#### Recruitment

DES patients consulting the Eye Clinic in Hadassah Medical Center with a complaint of dry eyes were approached for participation, as well as those who responded to an advertisement in Hadassah clinics and a local newspaper, or to e-mails addressed to Hadassah employees. Both prevalent and incident cases of SS were recruited in Oral Medicine, Eye, Rheumatology and Hematology clinics. The control group was recruited from healthy individuals who accompanied patients (not including blood relatives) to the hematology clinics. These individuals were approached in waiting areas and asked to participate [13].

The study was approved by the institutional Helsinki committee of Hadassah (study #: HMO-0409-13). All participants received an explanation on study aims and methods and signed an informed consent form prior enrollment. Data and blood samples were coded by study number.

### Blood count

All participants provided a 20ml blood sample. Anticoagulated samples were separated into plasma and cell fractions, within 24 hours of venipuncture, and mononuclear blood cells were frozen at -80°C. For DES and SS patients anticoagulated samples in 5 ml EDTA-containing tubes were also sent immediately for complete blood counts (*Coulter LH 750 hematology analyzer*, *Beckman*, *Brea*, *CA*, *USA)*

### Immune serology

Serum was tested for the presence of anti-nuclear antibodies (ANA), anti-SSA/anti-SSB, anti-ribonucleoprotein (RNP), anti-double stranded DNA (DS-DNA), and rheumatoid factor (RF) ("*Ofek* Laboratory service" Jerusalem, IL using the “*Immunodot”* assay qualitative-ELISA screening tool in an “autoimmunity screening panel” kit, *GenBlo*, San Diego, CA, USA).

Anti-SSA/Ro 52kD, anti-SSA/Ro 60kD, and anti-SSB/La were also quantified by multiplex serology following procedures described in detail previously [19–21] with newly developed antigens. Briefly, antigens SSA/Ro, and SSB/La were bacterially expressed in full length as recombinant proteins in fusion with N-terminal glutathione-S-transferase (GST) and C-terminally a small tagging epitope (tag). Each GST-X-tag fusion protein was bound and affinity-purified on a different bead set with glutathione surface and marked with a distinct internal fluorescent color (SeroMap, Luminex Corp., Austin, TX, USA). Bead sets loaded with the different antigens were mixed to form a suspension antigen array for presentation to a 1:1000 serum dilution. A Luminex xMAP (Luminex Corp., Austin, TX, USA) analyzer identified the bead set and simultaneously quantified bound serum IgA, IgM and IgG antibodies by a reporter fluorescent conjugate. The level of antibody response was given as median fluorescence intensity (MFI) on at least 100 beads per set. Net MFI values were generated by subtraction of bead-background and GST-tag background MFI values. For each antigen, qualitative cut-off levels for these autoantibodies (positive vs. negative) were chosen by visual inspection of cumulative histograms (percentile plots) [22–24] of the MFI values of all sera tested and preferring high specificity over high sensitivity [25] which resulted in 80 MFI for both SSA-Trim 52kD and SSA-Trove 60kD and, due to higher background reactivity, 200 MFI for SSB/La. This novel assay was validated in the current study against the “*Immunodot”* assay and clinical SS diagnostic criteria based on the AECG classification [11].

Immunoglobulins and C3 and C4 levels were tested using Immunoturbidimetric assay [“*COBAS INTEGRA*” system, “*COBAS-specific-proteins*” kit, “*Roche Diagnostics*”, Mannheim]. For budgetary reasons, testing for complement levels was only performed on a random sample of cases and controls. Five percent of the samples were analyzed blindly as duplicates for reproducibility.

### Sample size

Our study has 211 patients (SS+DES) and 211 controls, therefore, we have 80% power to detect an odds ratio (OR) as small as 2.2 fold, assuming 10% exposure in the controls^4^, and 5% error rate. For 91 patients and 211 controls, the corresponding detectable OR was 2.75 (*WinPepi* 11.63 software).

### Statistical analysis

Controls were frequency-matched to DES and SS cases by gender and age ±3 years. Normally distributed continuous variables were described using their means and standard deviation, and differences among groups were tested by t-test and one-way ANOVA. Non-normally distributed continuous variables were presented as medians with inter-quartile ranges and group differences were tested by Mann-Whitney and Kruskal-Wallis tests. Categorical variables were compared by *χ*^*2*^ test. Reproducibility of duplicate sample results was tested by Kappa score. Correlations between results of the two immune serology techniques were tested on paired samples by non-parametric McNemar test for dichotomous parameters and by Wilcoxon test for continuous crude levels, as well as grouped into quartiles. For graphical representation a log transformation was done for MFI levels. Validation indices calculated included sensitivity, specificity, positive predictive value (PPV) and negative predictive values (NPV). Multinomial regression models were used to compare DES, SS and controls, with adjustment for potential confounders. Pair-wise comparisons between diagnostic groups were performed using bivariate, then multivariate unconditional logistic regression models adjusting for age, gender and ethnicity. Results are presented as OR and 95% confidence intervals (CI). P<0.05 was considered statistically significant. Statistical analysis was done by *SPSS* 24.0 software (Chicago, IL 60606–6307).

## Results

### Clinical characteristics

The characteristics of study participants appear in Table 1. A female predilection in SS patients (9.2:1) compared to DES patients (6.8:1) was observed (P<0.001). Approximately half of the SS participants were classified as pSS and sSS, respectively. Among SS patients, clinical presentation was distributed rather equally between joint pain, dry mouth and dry eyes in this group. Two out of 120 DES patients were found to be positive for anti-SSA/B by immune serology (“Immunodot” assay), but were classified as DES rather than SS in the analysis since they fulfilled only 3 out of the 6 AECG criteria for SS (dry eye signs on Schirmer test, dry eye symptoms with OSDI score of 66 and 85 and positive anti-SSA/B)).

**Table 1:** Characteristics of the study population.

Characteristic	SS (n = 91)	DES (n = 120)	Control (n = 211)
**Mean age [SD] yrs**	57.58 [12.89]	52.04 [15.07]	54.57 [14.2]
**Age range yrs**	20–86	19–84	18–84
**Female gender n (%)**	84 (92.3)	82 (68.3)	168 (79.6)
**Ethnicity n (%)**			
East Europe	36 (39.6)	67 (55.8)	130 (61.6)
West Asia	22 (24.2)	20 (16.7)	35 (16.6)
North Africa	22 (24.2)	14 (11.7)	32 (15.2)
Israel	6 (6.5)	9 (7.5)	8 (3.8)
Mixed	5 (5.5)	10 (8.3)	6 (2.8)
**Education n (%)**			
≤ 12 years	38 (41.8)	29 (24.2)	58 (27.5)
> 12 years	53 (58.2)	91 (75.8)	153 (72.5)
**Secondary SS n (%)**	44 (48.6)	NA	NA
Rheumatoid arthritis (RA)	24 (26.6)	NA	NA
Systemic lupus erythematosus (SLE)	8 (8.8)	NA	NA
Thyroiditis	1 (1.1)	NA	NA
Mixed connective tissue disorder (MCTD)	2 (2.2)	NA	NA
Systemic sclerosis	2 (2.2)	NA	NA
Chronic hepatitis	1 (1.1)	NA	NA
Other	6 (6.6)	NA	NA
**Clinical presentation—SS/DES n (%)**			
Joint pain	35 (38.5)	NA	NA
Dry mouth	29 (31.9)	NA	NA
Dry eyes	27 (29.6)	120 (100)	NA

NA = not applicable.

There were significantly lower levels of lymphocytes and hemoglobin in SS compared to DES patients, however, the mean values across the groups were within the normal ranges (Table 2).

**Table 2:** Distribution of blood counts and immune serology positivity by group.

	Normal range	SS (n = 88)	DES (n = 103)		P^a^
		Mean [SD]	Mean [SD]		
White blood cell count (WBC)	4–10 (10E9/L)	6.44 [2.11]	6.95 [1.70]		0.067
Lymphocytes	1.5–4 (10E9/L)	**1.72 [0.66]**	**2.03 [0.51]**		**0.001**
Neutrophils	2–7.5 (10E9/L)	4.05 [1.76]	4.26 [1.41]		0.37
Hemoglobin (Males)	14–18 (g/dl)	**12.72 [1.93]**	**14.8 [1.10]**		**0.001**
(Females)	12–16 (g/dl)	**12.76 [1.15]**	**13.10 [1.10]**		**0.03**
Platelets	140–400 (10E9/L)	219 [51.30]	209 [54.10]		0.18
		**SS (n = 89)**	**DES (n = 102)**	**Odds Ratio (95%CI)**^**b**^	**P**^**c**^
		**Positive n (%)**	**Positive n (%)**		
Rheumatoid factor (RF)		**21 (23.6)**	**6 (5.9)**	**5.03 (1.78–14.21)**	**<0.001**
Anti-nuclear antibody (ANA)		**30 (33.7)**	**3 (2.9)**	**14.75 (4.09–53.17)**	**<0.001**
Anti-SSA/Anti-SSB		**31 (34.8)**	**2 (2.0)**	**20.97 (4.60–95.54)**	**<0.001**
Anti-ribonucleoprotein (RNP)		2 (2.2)	1 (1.0)	3.86 (0.26–55.80)	0.48
Double stranded (DS)-DNA		1 (1.1)	0 (0)	---	0.28

Positives to immune serology were tested by *"Immunodot"* assay.

^a^Based on t-test for independent samples.

^b^Tested by logistic regression; On multivariate analysis: Adjusted for age, gender and ethnicity.

^c^Chi-square test. WBC = white blood cell count; HGB = hemoglobin; OR = Odds ratio; CI = Confidence interval.

### Immune serology

In the control group, only three were positive for RF with negative serology to all other immune antigen components, therefore, we present the comparison only between SS and DES groups (Table 2). As noted, two DES patients (1.96%; 95%CI: 0.36–6.3) were found to be positive for anti-SSA/B. A 5- to 20-fold higher proportion of SS patients were positive for RF, ANA and anti-SSA/B antibodies compared to DES patients. In multivariate analysis adjusting for age, gender and ethnicity, we observed a strong positive association between positive RF (OR = 5.03, 95%CI: 1.78–14.21), ANA (OR = 14.75, 95%CI: 4.09–53.17) or a combination of anti-SSA/B (OR = 20.97, 95%CI: 4.60–95.54) autoantibodies in SS compared to DES patients (Table 2). Reproducibility was high with Kappa scores of 0.80 for RF and anti-SSA/B (P<0.01) and 0.62 for ANA (P = 0.03), comparing duplicate samples.

### “Immunodot” qualitative test

**Fig 1:**
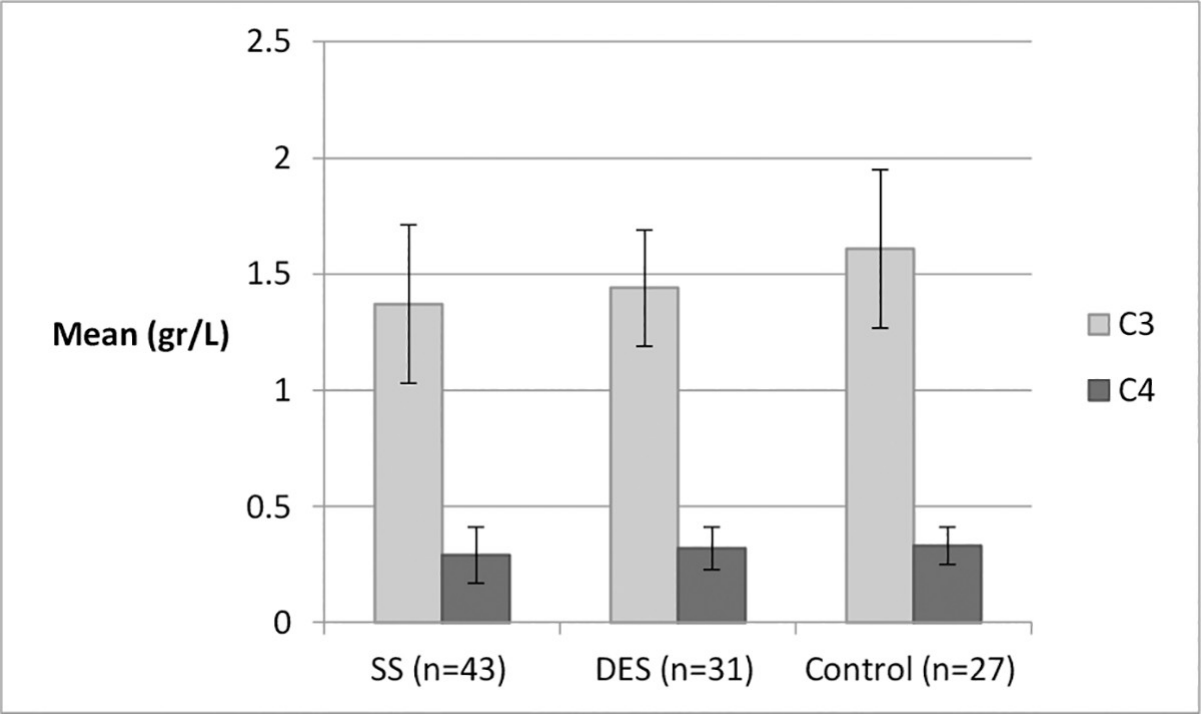
C3 and C4 distributions by group. *One-way ANOVA test; C3: **P = 0.009**, C4: P = 0.32. Normal ranges: C3: 0.9–0.18 gr/L; C4: 0.1–0.4 gr/L.

When comparing SS to DES groups based only on the “*Immunodot*” qualitative test results with adjustment for the same confounders, serum immunoglobulin IgG (gr/L) levels were found to be substantially higher in the SS group compared to DES and controls, with mean values within the normal range (Table 3). While C3 levels were lowest in SS patients, followed by DES and controls (P = 0.009); this pattern was not found for C4 levels (Fig 1).

**Table 3:** Comparison of immunoglobulin and autoantibodies levels by group.

Immunoglobulin (Normal ranges)	SS (n = 43)	DES (n = 31)	Control (n = 27)	P
	Median [IQR]	Median [IQR]	Median [IQR]	
**IgA** (0.7–4.0 gr/L)	2.67 [1.83–3.73]	2.49 [1.81–3.15]	2.71 [2.07–3.38]	0.69^**a**^
**IgG** (7.0–16.0 gr/L)	15.91 [12.19–20.02]	11.71 [10.27–13.52]	13.02 [11.41–16.73]	**<0.001**^**a**^
**IgM** (0.4–2.3 gr/L)	1.19 [0.86–1.84]	1.20 [0.80–1.64]	1.11 [0.81–1.75]	0.99^**a**^
**Autoantibody MFI values**	**SS (n = 91)**	**DES (n = 102)**	**Controls (n = 190)**	
	**Median [IQR]**	**Median [IQR]**	**Median [IQR]**	
Anti-SSA/Ro 52kD	36.0 [15.7–5203.7]	12.5 [5–23.2]	15.0 [7.0–29.5]	**<0.001**^**a**^
Anti-SSA/Ro 60kD	30.0 [15.5–214]	18.5 [9.7–36.5]	20 [12.0–35.5]	**<0.001**^**a**^
Anti-SSB/La	129 [47–3113.5]	40 [19–138.7]	69.0 [29.5–152.5]	**<0.001**^**a**^
	**SS Positive by MFI**^**c**^ **N (%)**	**DES Positive by MFI**^**c**^ **N (%)**	**Controls Positive by MFI**^**c**^ **N (%)**	
Anti-SSA/Ro 52kD (cut off: 80 MFI)	39 (42.8)	9 (8.8)	15 (7.8)	**<0.001**^**b**^
Anti-SSA/Ro 60kD (cut off: 80 MFI)	30 (32.9)	11 (10.7)	16 (8.4)	**<0.001**^**b**^
Anti-SSB/La (cut off: 200 MFI)	42 (46.1)	17 (16.6)	32 (16.8)	**<0.001**^**b**^
Positive for any anti-SSA or anti-SSB	54 (59.3)	31 (30.3)	6 (3.1)	**<0.001**^**b**^

^a^ Kruskal-Wallis test.

^b^ Chi-square test. IQR = inter quartile range. Autoantibodies were tested by glutathione-S-transferase (GST) capture ELISA in combination with fluorescent bead technology.

^c^ Positive by assay development.

### Comparison of the established to newly developed serologic technique

**Fig 2:**
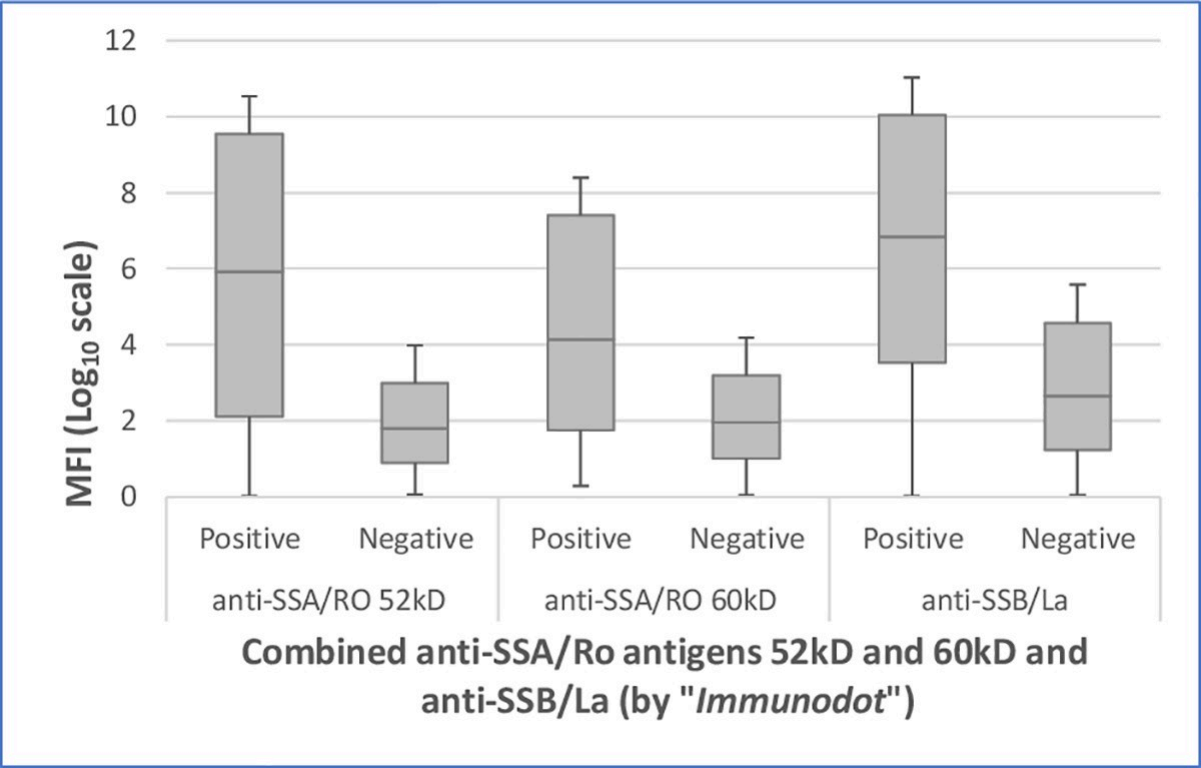
Comparison of positive and negative “*Immunodot*” results (for combined anti-SSA/Ro antigens 52kD and 60kD and anti-SSB/La) with log transformed median fluorescence intensity (MFI) levels for three antigens by multiplex serology (P<0.001). MFI = median fluorescence intensity.

Correlation results of the “*Immunodot”* qualitative assay and the newly developed multiplex serology revealed statistically significant associations for the dichotomous results (positive vs. negative) of these two methods for anti-SSB/La (McNemar test for paired samples: P<0.001) and anti-SSA/Ro 52kD (P = 0.02) and non-statistically significant for the anti-SSA/Ro 60kD (P = 0.09). However, when comparing the “*Immunodot”* assay results with the continuous MFI levels via the new technique, we observed significant strong associations for all three antigens, using the crude values and also after grouping into quartiles (Wilcoxon test for paired samples: P<0.001) (Fig 2).

### Multiplex serology method

The MFI levels of autoantibodies SSA/Ro 52kD, SSA/Ro 60kD and SSB/La using the multiplex serology method were found to be highest in the SS group, followed by the controls, with the lowest levels in the DES group (P<0.001) (Table 3). Furthermore, the proportion of patients found to be positive for these autoantigens with cut-off levels of 80 MFI for SSA and 200 MFI for SSB, revealed a higher proportion of positives to all three autoantigens in the SS group, followed by DES and controls (Table 3), similarly to the qualitative “*Immunodot*” array results. A pairwise categorical comparison also revealed highly significant differences between SS and DES (P<0.001) and between SS and controls (P<0.001), but not for DES compared to controls for all three autoantibodies (P = 0.21, 0.32 and 0.10 respectively). Stratified MFI levels by quartiles based on the control group levels showed about 75% of SS patients scored in the third and fourth quartiles, compared to DES patients in which only half had levels above the median for controls (S1 Table). Furthermore, a larger proportion of DES patients appeared to be in the lowest quartile compared to SS and controls.

Comparison of MFI levels among SS, DES and controls was done using a multinomial regression model. After adjusting for age, gender and ethnicity, an increased odds ratio was found for SS regarding presence of anti-SSA/Ro 52kD (OR = 2.05, 95%CI: 1.46–2.88) compared to controls, and a borderline inverse association was found between DES and anti-SSB/La (OR = 0.81, 95%CI: 0.65–1.00) compared to controls (Table 4). Other autoantibodies RNP and DS-DNA were rarely positive in the DES group (1% and 0% respectively) and the control group (0% for both) (data not shown).

**Table 4:** Multinomial regression of autoantibody titer quartiles comparing SS and DES vs. controls using multiplex serology.

Autoantibody	Control^a^	SS		DES	
	OR	OR	95% CI	OR	95% CI
**Univariate analysis**					
anti-SSA/Ro 52kD	1	**1.94**	**1.50–2.52**	0.84	0.68–1.05
anti-SSA/Ro 60kD	1	**1.38**	**1.10–1.73**	0.87	0.70–1.07
anti-SSB/La	1	**1.43**	**1.15–1.79**	0.82	0.66–1.01
**Multivariate analysis**^**b**^					
anti-SSA/Ro 52kD	1	**2.05**	**1.46–2.88**	0.83	0.67–1.04
anti-SSA/Ro 60kD	1	1.31	0.98–1.74	0.85	0.68–1.85
anti-SSB/La	1	1.22	0.92–1.62	**0.81**	**0.65–1.00**

^a^ Reference group.

^b^ Adjusted for age, gender and ethnicity. OR = Odds ratio; CI = Confidence interval. Tested by glutathione-S-transferase (GST) capture ELISA in combination with fluorescent bead technology.

Validation of the newly developed multiplex serology was done by comparing the MFI values to the clinically used “Immunodot” assay as a “gold-standard”. Seropositivity to any anti-SSA/B yielded a sensitivity of 1.0, specificity of 0.69, PPV of 0.35, and NPV of 1.0 (Table 5). In order to validate further the novel multiplex serology method we compared results, additionally, to the acceptable clinical criteria of AECG for diagnosis of Sjogren’s patients, which demonstrated high specificities to anti-SSA 52kD and 60kD of 0.91, and 0.87 to anti-SSB, yet rather low sensitivities (Table 5).

**Table 5:** Performance characteristics of dichotomized multiplex serology vs. Immunodot assay and AECG classification.

		*"Immunodot"* assay			Validity Indices
		Anti-SSA/B			
**Anti-SSA/Ro 52kD**		Positive N(%)	Negative N(%)	Total N(%)	Sen. = 0.87
***Multiplex serology (MFI)*** ^***a***^	Positive	27 (87)	20 (11)	47 (22)	Spec. = 0.89
	Negative	4 (13)	164 (89)	168 (78)	PPV = 0.57
P<0.001*	Total	31 (100)	184 (100)	215 (100)	NPV = 0.97
*Chi-Square test					
**Anti-SSA/Ro 60kD**					Sen. = 0.77
***Mulitplex serology (MFI)*** ^***a***^	Positive	24 (77)	16 (9)	40 (19)	Spec. = 0.91
	Negative	7 (23)	168 (91)	175 (81)	PPV = 0.60
P<0.001*	Total	31 (100)	184 (100)	215 (100)	NPV = 0.96
*Chi-Square test					
**Anti-SSB/La**					Sen. = 0.93
***Multiplex serology (MFI)*** ^***a***^	Positive	29 (94)	31 (17)	60 (28)	Spec. = 0.83
	Negative	2 (6)	153 (83)	155 (72)	PPV = 0.48
P<0.001*	Total	31 (100)	184 (100)	215 (100)	NPV = 0.98
*Chi-Square test					
**Any anti-SSA/B**					Sen. = 1.0
***Multiplex serology (MFI)*** ^***a***^	Positive	31 (100)	57 (30)	88 (41)	Spec. = 0.69
	Negative	0 (0)	127 (70)	127 (59)	PPV = 0.35
P<0.001*	Total	31 (100)	184 (100)	215 (100)	NPV = 1.0
*Chi-Square test					
		**AECG classification**			
**Anti-SSA/Ro 52kD**		Positive N(%)	Negative N(%)	Total N(%)	Sen. = 0.41
***Multiplex serology (MFI)*** ^***a***^	Positive	39 (42)	27 (9)	66 (17)	Spec. = 0.91
	Negative	54 (58)	275 (91)	329 (83)	PPV = 0.59
P<0.001*	Total	93 (100)	302 (100)	395 (100)	NPV = 0.83
*Chi-Square test					
**Anti-SSA/Ro 60kD**					Sen. = 0.32
***Multiplex serology (MFI)*** ^***a***^	Positive	30 (33)	26 (8)	56 (14)	Spec. = 0.91
	Negative	63 (67)	276 (92)	339 (86)	PPV = 0.53
P<0.001*	Total	93 (100)	302 (100)	395 (100)	NPV = 0.81
*Chi-Square test					
**Anti-SSB/La**					Sen. = 0.45
***Multiplex serology (MFI)*** ^***a***^	Positive	42 (45)	38 (13)	80 (20)	Spec. = 0.87
	Negative	51 (55)	264 (87)	315 (80)	PPV = 0.52
P<0.001*	Total	93 (100)	302 (100)	395 (100)	NPV = 0.83
*Chi-Square test					

^a^ Dichotomized by MFI levels. Sen. = Sensitivity; Spec. = Specificity; PPV = Positive Predictive Value; NPV = Negative Predictive Value. Cut-off level for Anti-SSA/Ro 52 and 60kD was 80 MFI, and for anti-SSB/La was 200 MFI.

A total of 13 participants were positive to any SSA/B by multiplex serology but negative by *Immunodot* with no signs or symptoms of dry eyes or mouth. In terms of demographic characteristics, theirmean age was 60.7±11.6, two were males (15%) and 11 females (85%), 6 (46%) were of East European ethnicity, 7 (54%) had a high school education or less and 4 (31%) had a first degree relative with other autoimmune disease. Further follow-up would be required to determine if they eventually develop autoimmune manifestations.

## Discussion

This is the first study in Israel to report the proportion of SS among patients with established DES. We found that 2% of patients presented with DES exhibited a combination of anti-SSA/Ro and anti-SSB/La autoantibodies, which form part of the diagnostic criteria for SS [11], in addition to their signs and symptoms of dry eyes. Worldwide, the prevalence of SS is ranges between 0.03% to 2.7% in various study populations using the same (AECG) or other diagnostic criteria (European, Copenhagen and San-Diego) [6].

When comparing hematological parameters in SS and DES patients, we observed lower levels of WBC, neutrophils, lymphocytes and hemoglobin were found in the former, but mean values were still within the normal ranges. Several studies have reported cytopenias including leukopenia, anemia, lymphopenia and neutropenia in SS patients [8,9,26,27]. However, these studies included only pSS cases who may have lower values of these hematologic parameters, while in our study we also included sSS cases, whereas no statistically significant difference was noted between these two groups regarding these parameters in our patient population.

Finally, we compared an established screening technique to the newly developed multiplex serology technique. These two serology methods were found to be highly correlated. The strong association between SS and autoantibody positivity found using both techniques was expected and is well known. However, results of MFI autoantibody levels have not previously been reported nor have immunoglobulin levels comparing healthy controls and patients with DES.

The validation indices comparing the newly developed multiplex serology to seropositivity of any anti-SSA/B compared to the Immunodot assay for combined presence of anti-SSA/B revealed a very high sensitivity and NPV, suggesting the new technique enables accurate distinction between AID cases and healthy controls.

Moreover, the seroprevalence of ANA in Israeli SS patients was previously reported by Friedman et al. to be 50%, RF 20%, anti-SSA/Ro 7% and anti-La 9% [28], while Kivity et al. reported that anti-SSA/Ro antibodies were detected in 62%, anti-SSB in 38%, ANA in 83%, anti-RNP in 11%, and DS-DNA in 27% [29]. These proportions are higher than the 34.8% for combination of anti SSA/B, 33.7% ANA, 2.2% anti-RNP, 1.1% DS-DNA antibodies and 23.6% RF, respectively, in our study.

The female:male ratio in SS (9.2:1) was, as expected, very high, as reported in many studies ranging from 8.4:1 [28] to 9.7:1 [29] in Israel, and up to 10:1 in a large pool of countries [30].

As in any case control study, associations do not infer causal relations, and potential biases exist. The temporal relation between hematologic disturbance and antibody appearance in SS is not clear, although it is likely that the latter predates the clinical diagnosis. Also, residual confounders, beyond those that were adjusted for, may have influenced the tested associations. The representativeness of controls for the general population may be limited, since this group included individuals accompanying patients to the clinic. SS and DES participants may also not be representative of all SS and DES populations considering the recruitment methods used. Following immune serology performed in the context of this study, two patients presenting with DES symptoms were found to have fulfilled the SS criteria after their recruitment. They were subsequently analyzed as SS patients. Another limitation includes variable storage time for SS and DES samples compared to controls, although all blood samples were immediately processed and stored at -80°C. The *Immunodot* combines serology of three antigens (anti SSA/Ro 52kD, 60kD and anti-SSB/La) limiting the ability to draw conclusions regarding individual autoantigens and their relationship with DES.

Strengths of this study includes the comparison of clinical and serologic parameters among three populations, two of which may present with similar symptoms. Additionally, we report the development of a new serologic technique, multiplex serology, which could be applicable to studies of large populations given its requirement for only small serum volumes, and high throughput potential. Further studies with larger sample sizes are needed to confirm serologic differences between SS and DES. It will be also intriguing to further investigate the correlation between serologic *Immunodot* methodology presented in this study and the EESDAI and ESSPRI of SS patients [31].

## Conclusions

Prevalence of SS among Israeli DES subjects was found to be similar to estimates of SS in population-based surveys of other countries. Hematologic and serological parameters, such as IgG, C3, seropositivity to ant-SSA/B, ANA and RF among SS patients differ from DES patients and healthy controls. A newly developed quantitative multiplex serology for relevant autoantigens seems to offer a valid and reliable quantitative technique to measure autoantibodies in such patients and may be applicable for use in large epidemiologic studies of SS and other autoimmune diseases.

## Supporting information

S1 Fig Cut-off levels of median fluorescence intensity (MFI) for each autoantigen. (DOCX)

S1 Table Autoantibody titer distributions by quartiles of controls by multiplex serology. (DOCX)

S1 Data (XLSX)

## References

[1] Courtin R, Pereira B, Naughton G, Chamoux A, Chiambaretta F, Lanhers C, Dutheil F. Prevalence of dry eye disease in visual display terminal workers: a systematic review and meta-analysis. BMJ Open. 2016;6(1). 10.1136/bmjopen-2015-009675 26769784 PMC4735196

[2] The definition and classification of dry eye disease: report of the Definition and Classification Subcommittee of the International Dry Eye WorkShop (2007). Ocul Surf. 2007;5(2):75–92. 10.1016/s1542-0124(12)70081-2 17508116

[3] Moss SE, Klein R, Klein BEK. Incidence of dry eye in an older population. Arch Ophthalmol. 2004;122(3):369–73. 10.1001/archopht.122.3.369 15006852

[4] Galor A, Feuer W, Lee DJ, Florez H, Carter D, Pouyeh B, et al Prevalence and risk factors of dry eye syndrome in a United States Veterans Affairs population. Am J Ophthalmol [Internet]. 2011;152(3):377–84. 10.1016/j.ajo.2011.02.026 21684522 PMC4113967

[5] Shah S, Jani H. Prevalence and associated factors of dry eye: Our experience in patients above 40 years of age at a Tertiary Care Center. Oman J Ophthalmol [Internet]. 2015;8(3):151 10.4103/0974-620X.169910 26903719 PMC4738658

[6] Patel R, Shahane A. The epidemiology of Sjögren’s syndrome. Clin Epidemiol. 2014;6(1):247–55. 10.2147/CLEP.S47399 25114590 PMC4122257

[7] Chen W, Cao H, Lin J, Olsen N, Zheng SG. Biomarkers for Primary Sjögren’s Syndrome. Genomics Proteomics Bioinformatics [Internet]. 2015;13(4):219–23. 10.1016/j.gpb.2015.06.002 26362815 PMC4610966

[8] Baimpa E, Dahabreh IJ, Voulgarelis M, Moutsopoulos HM. Hematologic manifestations and predictors of lymphoma development in primary Sjögren syndrome: clinical and pathophysiologic aspects. Medicine (Baltimore). 2009;88(5):284–93. 10.1097/MD.0b013e3181b76ab5 19745687

[9] Voulgarelis M, Ziakas PD, Papageorgiou A, Baimpa E, Tzioufas AG, Moutsopoulos HM. Prognosis and outcome of non-Hodgkin lymphoma in primary Sjögren syndrome. Medicine (Baltimore) [Internet]. 2012;91(1):1–9. 10.1097/MD.0b013e31824125e4 22198497

[10] Turner MD. Salivary gland disease in Sjögren’s syndrome: sialoadenitis to lymphoma. Oral Maxillofac Surg Clin North Am. 2014;26(1):75–81. 10.1016/j.coms.2013.09.006 24287195

[11] Vitali C, Bombardieri S, Jonsson R, Moutsopoulos HM, Alexander EL, Carsons SE, et al Classification criteria for Sjögren’s syndrome: a revised version of the European criteria proposed by the American-European Consensus Group. Ann Rheum Dis. 2002;61(6):554–8. 10.1136/ard.61.6.554 12006334 PMC1754137

[12] Shiboski CH, Shiboski SC, Seror R, Rasmussen A, Scofield H, Vitali C, et al 2016 ACR-EULAR Classification Criteria for primary Sjögren’s Syndrome: A Consensus and Data-Driven Methodology Involving Three International Patient Cohorts. Arthritis Rheumatol. 2017;69(1):35–45. 10.1002/art.39859 27785888 PMC5650478

[13] Kleinstern G, Abu Seir R, Perlman R, Khatib A, Abdeen Z, Elyan H, et al Ethnic variation in medical and lifestyle risk factors for B cell non-Hodgkin lymphoma: A case-control study among Israelis and Palestinians. PLoS One. 2017;12(2):1–12. 10.1371/journal.pone.0171709 28196110 PMC5308607

[14] Ben-Eli H, Aframian DJ, Ben-chetrit E, Mevorach D, Kleinstern G, Paltiel O, et al Shared Medical and Environmental Risk Factors in Dry Eye Syndrome, Sjogren’s Syndrome, and B-Cell Non-Hodgkin Lymphoma: A Case-Control Study. J Immunol Res. 2019, 019 Jan 21;2019:9060842. 10.1155/2019/9060842 eCollection 2019. 30805374 PMC6360537

[15] Schiffman RM, Christianson MD, Jacobsen G, Hirsch JDRB. Reliability and Validity of the Ocular Surface Disease Index. Arch Ophthalmol. 2000;118(5):615–21. 10.1001/archopht.118.5.615 10815152

[16] McDonald M, Patel DA, Keith MS, Snedecor SJ. Economic and Humanistic Burden of Dry Eye Disease in Europe, North America, and Asia: A Systematic Literature Review. Ocul Surf. 2016;14(2):144–67. 10.1016/j.jtos.2015.11.002 26733111

[17] Vehof J, Smitt-kamminga NS, Kozareva D, Nibourg SA, Hammond CJ. Clinical Characteristics of Dry Eye Patients With Chronic Pain Syndromes. Am J Ophthalmol. 2016;166:203–4. 10.1016/j.ajo.2016.03.023 27090221

[18] Sweeney DF, Millar TJ, Raju SR. Tear film stability: A review. Exp Eye Res. 2013;117:28–38. 10.1016/j.exer.2013.08.010 23973716

[19] Waterboer T, Sehr P, Pawlita M. Suppression of non-specific binding in serological Luminex assays. J Immunol Methods. 2006;309(1–2):200–4. 10.1016/j.jim.2005.11.008 16406059

[20] Waterboer T, Sehr P, Michael KM, Franceschi S, Nieland JD, Joos TOTM, et al Multiplex human papillomavirus serology based on in situ-purified glutathione S-transferase fusion proteins. Clin Chem. 2005;51(10):1845–53. 10.1373/clinchem.2005.052381 16099939

[21] Butt J, Jenab M, Willhauck-Fleckenstein M, Michel A, Pawlita M, Kyrø C, et al Prospective evaluation of antibody response to *Streptococcus gallolyticus* and risk of colorectal cancer. Int J Cancer [Internet]. 2018 10.1002/ijc.31283 29377173

[22] Dana E, Rollison, Giuliano Anna R., Messina Jane L, Fenske Neil A., Cherpelis Basil S., et al Case–control Study of Merkel Cell Polyomavirus Infection and Cutaneous Squamous Cell Carcinoma. Cancer Epidemiol Biomarkers Prev. 2012;21(1):74–81. 10.1158/1055-9965.EPI-11-0764 22016472 PMC4543303

[23] Michael KM, Waterboer T, Sehr P, Rother A, Reidel U, Boeing H, et al Seroprevalence of 34 human papillomavirus types in the German general population. PLoS Pathog. 2008;4(6). 10.1371/journal.ppat.1000091 18566657 PMC2408730

[24] Migchelsen SJ, Martin DL, Southisombath K, Turyaguma P, Heggen A, Rubangakene PP, et al Defining Seropositivity Thresholds for Use in Trachoma Elimination Studies. PLoS Negl Trop Dis. 2017;11(1):1–19. 10.1371/journal.pntd.0005230 28099433 PMC5242428

[25] Brenner N, Mentzer AJ, Butt J, Michel A, Prager K, Brozy J, et al Validation of Multiplex Serology detecting human herpesviruses 1–5. PLoS One. 2018;96:1–20. 10.1371/journal.pone.0209379 30589867 PMC6307738

[26] Quartuccio L, Isola M, Baldini C, Priori R, Bartoloni Bocci E, Carubbi F, et al Biomarkers of lymphoma in Sjögren’s syndrome and evaluation of the lymphoma risk in prelymphomatous conditions: Results of a multicenter study. J Autoimmun [Internet]. 2014;51:75–80. 10.1016/j.jaut.2013.10.002 24231556

[27] Solans-Laqué R, López-Hernandez A, Angel Bosch-Gil J, Palacios A, Campillo M, Vilardell-Tarres M. Risk, Predictors, and Clinical Characteristics of Lymphoma Development in Primary Sjögren’s Syndrome. Semin Arthritis Rheum. 2011;41(3):415–23. 10.1016/j.semarthrit.2011.04.006 21665245

[28] Friedman JA, Miller EB, Green L, Huszar M, Schattner A. A community-based cohort of 201 consecutive patients with primary Sjögren’s syndrome in Israel: Ashkenazi patients compared with those of Sephardic descent. Clin Exp Rheumatol. 2006;24(3):274–80. 16870094

[29] Kivity S, Arango MT, Ehrenfeld M, Tehori O, Shoenfeld Y, Anaya JM, et al Infection and autoimmunity in Sjogren’s syndrome: A clinical study and comprehensive review. J Autoimmun [Internet]. 2014;51:17–22. 10.1016/j.jaut.2014.02.008 24637076

[30] Ramos-casals M, Brito-zerón P, Kostov B, Sisó-almirall A, Bosch X, Buss D, et al Google-driven search for big data in autoimmune geoepidemiology: Analysis of 394, 827 patients with systemic autoimmune diseases. Autoimmun Rev. 2015;14(8):670–9. 10.1016/j.autrev.2015.03.008 25842074

[31] Seror R, Bootsma H, Saraux A, Bowman SJ, Theander E, Brun JG, et al Defining disease activity states and clinically meaningful improvement in primary Sjögren’s syndrome with EULAR primary Sjögren’s syndrome disease activity (ESSDAI) and patient-reported indexes (ESSPRI) Raphaèle. Ann Rheum Dis. 2016;75(2):382–9. 10.1136/annrheumdis-2014-206008 25480887

