# Supplementary figures and images for: Serological and hematological characteristics of Sjogren’s syndrome and dry eye syndrome patients using a novel immune serology technique
Source: PLoS One. 2020 Dec 31;15(12):e0244712. doi: 10.1371/journal.pone.0244712 (PMC7774976; doi:10.1371/journal.pone.0244712)

**Figure 1:** Cut-off levels of median fluorescence intensity (MFI) for each autoantigen.


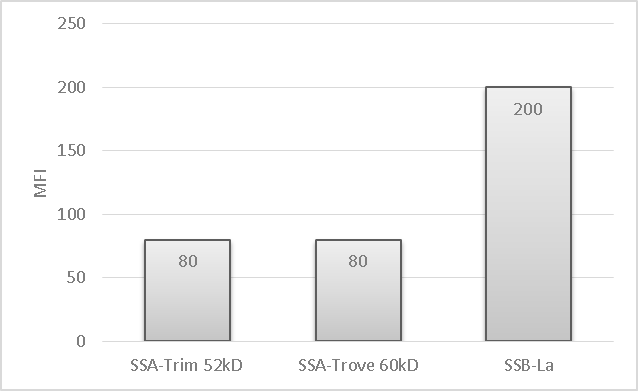

Supplement: S1 Fig — (DOCX) [file pone.0244712.s001.docx]
