# Supplementary material for: Serological and hematological characteristics of Sjogren’s syndrome and dry eye syndrome patients using a novel immune serology technique
Source: PLoS One. 2020 Dec 31;15(12):e0244712. doi: 10.1371/journal.pone.0244712 (PMC7774976; doi:10.1371/journal.pone.0244712)
Supplement: S1 Table — (DOCX) [file pone.0244712.s002.docx]

| **Table 1: Autoantibody titer distributions by quartiles of controls by multiplex serology** | | | | |
| --- | --- | --- | --- | --- |
| **Autoantibody**  **(Quartile borders in MFI)** | **Control^a^**  (n=190)  N (%) | **DES**  (n=102)  N (%) | **SS**  (n=93)  N (%) | **P^b^** |
| **anti-SSA 52kD** |  |  |  | **<0.001** |
| Q1 (<7) | 42 (22.1) | 33 (32.4) | 4 (4.3) |  |
| Q2 (7-15) | 51 (26.8) | 21 (20.6) | 16 (17.2) |  |
| Q3 (15-29) | 47 (24.7) | 27 (26.5) | 20 (21.5) |  |
| Q4 (>29) | 50 (26.4) | 21 (20.5) | 53 (57.0) |  |
| **anti-SSA 60kD** |  |  |  | **<0.001** |
| Q1 (<12) | 44 (23.2) | 35 (34.3) | 13 (14.0) |  |
| Q2 (12-20) | 45 (23.7) | 20 (19.6) | 19 (20.4) |  |
| Q3 (20-35) | 52 (27.4) | 20 (19.6) | 16 (17.2) |  |
| Q4 (>35) | 49 (25.7) | 27 (26.5) | 45 (48.4) |  |
| **anti-SSB** |  |  |  | **<0.001** |
| Q1 (<29) | 46 (24.2) | 41 (40.2) | 17 (18.3) |  |
| Q2 (29-69) | 52 (27.4) | 19 (18.6) | 11 (11.8) |  |
| Q3 (69-152) | 44 (23.2) | 19 (18.6) | 20 (21.5) |  |
| Q4 (>152) | 48 (25.2) | 23 (22.4) | 45 (48.4) |  |
| ^a^ Reference group. ^b^ Chi-square test | | | | |

**protein databank accession number showing the cDNA sequences that were used to produce the recombinant proteins used to prepare the multi-plex:**

SSA-Ro50_TRIM21 🡪  AAU89982 ([https://www.ncbi.nlm.nih.gov/protein/AAU89982.1?report=genbank&log$=prottop&blast_rank=1&RID=U2AT1CSJ016](https://urldefense.proofpoint.com/v2/url?u=https-3A__www.ncbi.nlm.nih.gov_protein_AAU89982.1-3Freport-3Dgenbank-26log-24-3Dprottop-26blast-5Frank-3D1-26RID-3DU2AT1CSJ016&d=DwMGaQ&c=bw8D93A58cSgXedRoUYFaNvg5iAVftQZmTcV3G3662E&r=SOJfK7z5SDCcQXlUe00Hrhi7f7IWsFwf68pB0kypoAg&m=wnyLy8wfngx49lNdtLoM2218GdK4s5KdJRwHsG6FBM4&s=7Jl5lhW6ZqTsNJGp2Sz3pSi2r_i-dHMcpJiW6ZCA0s4&e=))

SSA-Ro60_TROVE2 🡪 NP_001035828 ([https://www.ncbi.nlm.nih.gov/protein/NP_001035828.1?report=genbank&log$=protalign&blast_rank=1&RID=U2B7AJSB016](https://urldefense.proofpoint.com/v2/url?u=https-3A__www.ncbi.nlm.nih.gov_protein_NP-5F001035828.1-3Freport-3Dgenbank-26log-24-3Dprotalign-26blast-5Frank-3D1-26RID-3DU2B7AJSB016&d=DwMGaQ&c=bw8D93A58cSgXedRoUYFaNvg5iAVftQZmTcV3G3662E&r=SOJfK7z5SDCcQXlUe00Hrhi7f7IWsFwf68pB0kypoAg&m=wnyLy8wfngx49lNdtLoM2218GdK4s5KdJRwHsG6FBM4&s=6qrgXubumtPS73jLlarXv-lFjtqp2O9F7fjjimxYHro&e=))

SSB-La 🡪  NP_003133 ([https://www.ncbi.nlm.nih.gov/protein/NP_003133.1?report=genbank&log$=protalign&blast_rank=1&RID=U2B65KP0013](https://urldefense.proofpoint.com/v2/url?u=https-3A__www.ncbi.nlm.nih.gov_protein_NP-5F003133.1-3Freport-3Dgenbank-26log-24-3Dprotalign-26blast-5Frank-3D1-26RID-3DU2B65KP0013&d=DwMGaQ&c=bw8D93A58cSgXedRoUYFaNvg5iAVftQZmTcV3G3662E&r=SOJfK7z5SDCcQXlUe00Hrhi7f7IWsFwf68pB0kypoAg&m=wnyLy8wfngx49lNdtLoM2218GdK4s5KdJRwHsG6FBM4&s=SHihA-R50V-TZ3eqvg71zhyqsHp8eGtLxnjnT8k4Zck&e=))

Note the sequences were codon-optimized for expression in E.coli
